# Serum 25-hydroxyvitamin D as a predictive biomarker of clinical outcomes in patients with primary membranous nephropathy

**DOI:** 10.3389/fnut.2023.1171216

**Published:** 2023-04-25

**Authors:** Suyan Duan, Si Chen, Fang Lu, Meng Zhou, Ling Jiang, Chen Chen, Luhan Geng, Rui Sun, Yili Xu, Zhimin Huang, Chengning Zhang, Bo Zhang, Huijuan Mao, Changying Xing, Yanggang Yuan

**Affiliations:** Department of Nephrology, The First Affiliated Hospital of Nanjing Medical University, Nanjing Medical University, Nanjing, China

**Keywords:** 25 (OH) vitamin D, primary membranous nephropathy, anti-phospholipase A2 receptor antibody, proteinuria, predictor

## Abstract

**Background:**

Primary membranous nephropathy (PMN) is an immune-related disease with increased morbidity and the most common cause of adult nephrotic syndrome (NS). The serum 25-hydroxyvitamin D [25(OH)D)], a biomarker of vitamin D (VD) status, tends to decline in patients with kidney disease. However, the relationship between 25(OH)D and PMN is still unclear. Therefore, this study aims to clarify the association between 25(OH)D and disease severity and therapy response of PMN.

**Methods:**

A total of 490 participants diagnosed with PMN by biopsy from January 2017 to April 2022 were recruited at the First Affiliated Hospital of Nanjing Medical University. The correlations between baseline 25(OH)D and manifestations of nephrotic syndrome (NS) or seropositivity of anti-PLA2R Ab were confirmed by univariate and multivariate logistic analyses. Spearman’s correlations were used to examine the associations between baseline 25(OH)D and other clinical parameters. In the follow-up cohort, Kaplan-Meier analysis was used to assess remission outcomes among groups with low, medium, and high levels of 25(OH)D. Furthermore, the independent risk factors for non-remission (NR) were explored by COX regression analysis.

**Results:**

At baseline, 25(OH)D was negatively related to 24-h urinary protein and serum anti-PLA2R Ab. The lower level of baseline 25(OH)D was associated with an increased risk for the incidence of NS in PMN (model 2, OR 6.8, 95% CI 4.4, 10.7, *P* < 0.001) and seropositivity of anti-PLA2R Ab (model 2, OR 2.4, 95% CI 1.6, 3.7, *P* < 0.001). Furthermore, the lower level of 25(OH)D during follow-up was demonstrated as an independent risk factor for NR even after adjusting age, gender, MBP, 24 h UP, serum anti-PLA2R Ab, serum albumin, and serum C3 [25(OH)D (39.2–62.3 nmol/L): HR 4.90, 95% CI 1.02, 23.53 *P* = 0.047; 25(OH)D < 39.2 nmol/L: HR 17.52, 95% CI 4.04, 76.03 *P* < 0.001); vs. 25(OH)D ≥ 62.3 nmol/L]. The Kaplan-Meier survival analysis also demonstrated that the higher level of follow-up 25(OH)D had a higher possibility of remission than the lower one (log-rank test, *P* < 0.001).

**Conclusion:**

Baseline 25(OH)D was significantly correlated with nephrotic proteinuria and seropositivity of anti-PLA2R Ab in PMN. As an independent risk factor for NR, a low level of 25(OH)D during follow-up might serve as a prognostic tool for sensitively identifying cases with a high probability of poor treatment response.

## Introduction

Primary membranous nephropathy (PMN) is a podocyte-related disease characterized by adult patients with nephrotic syndrome (NS), with an increasing incidence rate in recent years (1). Most patients suffered from heavy proteinuria, hypoproteinemia, and high risks for infection. Phospholipase A2 receptor (PLA2R), a podocyte-targeted antigen, accounts for the pathogenesis in 70–80% of patients with PMN (2). Serum anti-PLA2R antibodies (Ab) were widely used in diagnosis, monitoring treatment, and evaluating prognosis in clinical practice (2–5).

Vitamin D (VD), a key substance for bone growth and calcium homeostasis, has been demonstrated to play an important role beyond the musculoskeletal system in recent years (6). The majority of VD was derived from 7-dehyrocholesterol in the skin, and a small amount was obtained from a daily diet. 25-hydroxyvitamin D [25(OH)D] was the metabolic product of VD in the liver and was abundant in circulation, acting as a reliable biomarker for VD status (7). A low level of 25(OH)D was not only closely related to higher cardiovascular mortality (8) but was also common in proteinuric kidney disease (7). Besides, 25(OH)D was converted into the active form of VD [1,25-dihydroxyvitamin (1,25 (OH)2 D3)] by the cytochrome P450 enzyme, 1-alpha-hydroxylase (CYP27B1), in the kidney and other tissues (6, 7). Hence, low serum 25(OH)D concentrations contributed to reduced 1,25 (OH)2 D3 levels by providing less substrate for conversion (9). 1,25 (OH)2 D3 has been shown to play a role in tumor cell metabolism *via* regulating cell proliferation, apoptosis, and inflammation and to participate in autoimmune diseases by regulating both adaptive and innate immunity (6, 10–12). More importantly, in chronic kidney disease (CKD), 1,25 (OH)2 D3 was associated with creatinine clearance and disease progression (13, 14). Activated VD has been determined to enhance the antiproteinuric effect of renin-angiotensin-aldosterone system (RAAS) inhibitors in IgA nephropathy, lupus nephritis (LN), and as a renoprotector delaying the onset of diabetic kidney disease (DKD) (6, 14). However, it remains largely unclear whether VD is associated with disease activity and treatment response in patients with PMN.

The current study aimed to investigate the association of serum 25(OH)D level with clinical parameters of disease activity and the predictive ability of risk stratification. Furthermore, the predictive value of follow-up 25(OH)D on the treatment response of patients with PMN who had NS was also assessed to reinforce the point.

## Materials and methods

### Participant selection

**FIGURE 1:**
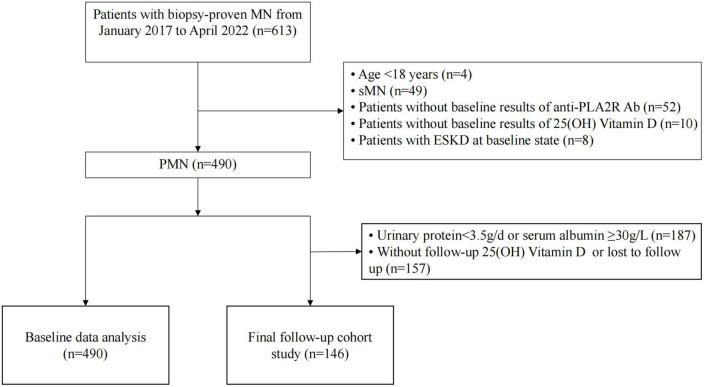
Flowchart of study participants. PMN, primary membranous nephropathy; sMN, secondary membranous nephropathy; anti-PLA2R Ab, anti-phospholipase A2 receptor antibody; 25(OH)D, 25-hydroxyvitamin D; ESKD, end-stage kidney disease.

Patients diagnosed with PMN by renal biopsy at the First Affiliated Hospital of Nanjing Medical University from January 2017 to April 2022 were retrospectively reviewed. The inclusion criteria were as follows: (1) age ≥ 18 years; and (2) newly diagnosed MN confirmed by renal biopsy. The exclusion criteria were as follows: (1) secondary to other diseases, including hepatitis B virus (HBV) and other infections, tumors, and autoimmune diseases; (2) patients with incomplete baseline data, such as serum anti-PLA2R Ab and 25(OH)D; and (3) patients with end-stage kidney disease (ESKD) (eGFR ≤ 15 ml/min/1.73 m^2^ or who received renal replacement therapy) at baseline. A previous study was referred to as the theoretical or reference basis for the selection criteria (15). In addition, as suggested by the KDIGO 2021 guidelines on glomerular diseases, patients with 24-h urinary protein >3.5 g were stratified into at least a moderate-risk group. Therefore, they were closely monitored and constituted the follow-up cohort in the current study (16). In total, the follow-up cohort was composed of 146 patients with NS, a follow-up time >6 months, and complete follow-up records, including 25(OH) D levels (Figure 1).

### Data collection

All demographic data (sex and age) and clinical data, including systolic (SBP) and diastolic blood pressure (DBP), serum anti-PLA2R Ab, 24-h urinary protein, serum albumin, serum 25 (OH)D, total cholesterol (TC), triglyceride (TG), high-density lipoprotein cholesterol (HLD-C), low-density lipoprotein cholesterol (LDL-C), serum calcium, serum phosphorus, serum creatinine, estimated glomerular filtration rate (eGFR), serum uric acid (UA), serum immunoglobulin G (IgG), serum immunoglobulin A (IgA), serum immunoglobulin M (IgM), serum complement 3 (C3), serum complement 4 (C4), serum parathyroid hormone, the history of hypertension, diabetes mellitus (DM), infection, and the therapeutic regimen (glucocorticoids, immunosuppressive agents, angiotensin converting enzyme inhibitor or angiotensin receptor inhibitor (ACEI/ARB), and VD supplementary treatment), were collected from medical records at the time of renal biopsy. Clinical response to treatment was also recorded for each patient. These patients were followed up for at least 6 months. At the most recent follow-up, 25 (OH)D, 24-h urinary protein, eGFR, and the therapeutic regimen (glucocorticoids, immunosuppressive agents, and ACEI/ARB) were recorded. Mean blood pressure (MBP) was calculated as DBP plus one-third of the difference between SBP and DBP. eGFR was calculated according to Chronic Kidney Disease Epidemiology Collaboration (CKD-EPI) equation (17). The serum 25(OH)D level was determined by electrochemiluminescence immunoassay according to the manufacturer’s instructions (Roche Diagnostic GmBH, Germany). △25(OH)D was the difference of 25(OH)D between baseline and follow-up.

### Treatment and follow-up

The therapeutic regimens (use of glucocorticoids, immunosuppressive agents, and ACEI/ARB) and the definitions of remission complied with the KDIGO (Kidney Disease: Improving Global Outcomes) guidelines for glomerulonephritis. According to the Kidney Disease Outcomes Quality Initiative (KDOQI) guidelines, patients with MN who had VD insufficiency (<30 ng/ml) initiated VD supplementation (18). During follow-up, treatment success was evaluated after at least 6 to 12 weeks by measuring serum 25(OH)D to target concentrations of 30–50 ng/ml (75 to 125 nmol/L) (19).

Complete remission (CR) was defined as urinary protein excretion of <0.3 g/day with a stable GFR. Partial remission (PR) was defined as urinary protein excretion of <3.5 g/day and at least a 50% reduction from baseline values with a stable GFR. Otherwise, the outcome was defined as non-remission or non-response (NR). ESKD was defined as the initiation of maintenance dialysis or kidney transplantation.

### Statistical analysis

Mean and standard deviation (SD), median with interquartile range (IQR), or frequencies with percentages were employed separately to describe data distribution as appropriate. Comparisons between groups were performed using one-way analysis of variance (ANOVA), Student’s *t*-test, Kruskal–Wallis test, Wilcoxon rank-sum test, or χ^2^ test as appropriate. Correlations between baseline 25(OH)D and clinical parameters were performed using Spearman’s correlation. Receiver-operating characteristic (ROC) curve analysis was used to explore the predictive value of the 25(OH)D level at baseline and follow-up time. Logistic regression analysis was used to assess the association between baseline 25(OH)D and patients with NS or serum anti-PLA2R Ab level. Univariate and multivariate Cox proportional hazards models were employed to investigate the risk factors for NR in the follow-up cohort. Moreover, Kaplan–Meier analysis and the log-rank test were used to compare the remission rate between groups with different levels of follow-up 25(OH) D. All values were analyzed using IBM SPSS Statistics version 26.0 and R version 4.0.2, and a two-tailed *P*-value of <0.05 was considered significant.

## Results

### Comparisons of baseline parameters according to different levels of urinary protein

A total of 490 patients diagnosed with PMN by biopsy were divided into three groups according to levels of 24-h urinary protein (UP). Patients with massive proteinuria (24 h UP > 8 g) were assigned to group 3, and those with mild proteinuria (24 h UP < 3.5g) were assigned to group 1. As shown in Table 1, among 490 participants, 301 (61.4%) were men, and the median age was 54.0 (IQR 42.0, 63.0) years. At the time of biopsy, patients with positive serum anti-PLA2RAb (≥ 20 RU/ml) accounted for 60.2% of the total cohort. The median baseline 25(OH)D was 17.7 (IQR 9.8, 28.5) nmol/L, and 451 (92.0%) patients received VD supplements. Moreover, levels of SBP, DBP, serum albumin, baseline 25(OH)D, TC, LDL-C, serum calcium, serum creatinine, eGFR, serum IgG, serum C4, the ratio of hypertension, and the number of VD receivers were significantly different between any two of the three groups (*P* < 0.05). Meanwhile, the titers of serum anti-PLA2R Ab, TG, and serum C3, the proportion of male patients and positive serum anti-PLA2R Ab, and the use of glucocorticoids and immunosuppressive agents were also statistically different among the three groups. No significant difference was observed between Group 2 and Group 3 (*P* < 0.05). However, there was no statistical difference in age and other clinical parameters among the three groups, including HDL-C, serum phosphorus, serum uric acid, serum IgA, serum IgM, the proportion of DM and infection, and the use of ACEI/ARB (*P* > 0.05).

**TABLE 1:** Baseline characteristics of patients with different levels of proteinuria.

	Total	Urinary protein (g/day)			*P*-value
**Parameter**	***n* = 490**	**<3.5 (*n* = 177)**	**3.5–8.0 (*n* = 194)**	**>8.0 (*n* = 119)**	
**Demographic characteristics**					
Male sex, no. (%)	301 (61.4%)	84 (47.5%)	128 (66.0%)	89 (74.8%)	**<0.001^#^**
Age, years	54.0 (42.0,63.0)	52.0 (40.5,60.0)	55.0 (44.0,65.0)	54.0 (40.0,63.0)	0.063
**Clinical characteristics**					
SBP, mmHg	131.0 (121.0,142.25)	126.0 (116.0,138.0)	131.0 (121.0,145.0)	137.0 (127.0,149.0)	<0.001*
DBP, mmHg	83.0 (75.0,90.0)	80.0 (72.0,87.0)	83.0 (76.0,92.0)	87.0 (80.0,95.0)	<0.001*
**Laboratory characteristics**					
Anti-PLA2R Ab, RU/ml	35.90 (4.31,145.47)	9.64 (1.59,51.93)	72.09 (13.99,188.51)	74.69 (15.39,216.20)	**<0.001^#^**
Serum anti-PLA2R Ab positive, no. (%)	295 (60.2%)	71 (40.1%)	136 (70.1%)	88 (73.9%)	**<0.001^#^**
Albumin, g/L	23.9 ± 7.0	29.4 ± 6.60	22.0 ± 5.1	19.1 ± 4.4	<0.001*
Baseline 25 (OH)D, nmol/L	17.7 (9.8,28.5)	27.4 (18.9,41.2)	16.0 (9.4,24.5)	9.9 (3.8,16.2)	<0.001*
TC, mmol/L	6.94 (5.57,8.55)	5.76 (4.66,7.26)	7.22 (5.99,8.94)	8.14 (6.74,9.72)	<0.001*
TG, mmol/L	1.85 (1.34,2.74)	1.52 (1.13,2.25)	2.04 (1.48,2.86)	2.41 (1.61,3.37)	**<0.001^#^**
HDL-C, mmol/L	1.36 (1.12,1.63)	1.33 (1.08,1.63)	1.39 (1.12,1.64)	1.39 (1.16,1.73)	0.252
LDL-C, mmol/L	4.32 (3.35,5.49)	3.50 (2.79,4.58)	4.68 (3.57,5.60)	5.14 (4.13,6.25)	<0.001*
Serum calcium, mmol/L	1.97 ± 0.17	2.07 ± 0.14	1.94 ± 0.13	1.86 ± 0.19	<0.001*
Serum phosphorus, mmol/L	1.18 ± 0.21	1.18 ± 0.24	1.19 ± 0.19	1.19 ± 0.22	0.761
Serum creatinine, μmol/L	69.10 (56.58,83.98)	60.80 (50.85,73.60)	70.75 (60.20,84.70)	82.80 (65.30,102.50)	<0.001*
eGFR, ml/min/1.73 m^2^	99.33 (83.97,111.40)	105.4 (95.8,116.6)	97.9 (82.6,107.2)	91.00 (67.62,105.72)	<0.001*
Uric acid, μmol/L	373.5 ± 104.6	362.9 ± 103.7	382.5 ± 103.3	374.8 ± 107.4	0.169
Serum IgG, g/L	5.65 (3.87,7.58)	7.25 (5.32,9.42)	5.36 (4.04,6.93)	4.19 (2.85,5.92)	<0.001*
Serum IgA, g/L	2.11 (1.60,2.80)	2.17 (1.65,2.93)	2.07 (1.53,2.73)	2.13 (1.55,2.79)	0.303
Serum IgM, g/L	1.03 (0.69,1.41)	1.08 (0.73,1.48)	1.10 (0.73,1.40)	0.93 (0.65,1.39)	0.387
Serum C3, g/L	1.0 ± 0.2	0.9 ± 0.2	1.0 ± 0.2	1.0 ± 0.2	**0.001^#^**
Serum C4, g/L	0.24 (0.20,0.31)	0.22 (0.18,0.26)	0.25 (0.20,0.32)	0.28 (0.23,0.32)	<0.001*
**Comorbid disease**					
Hypertension no. (%)	215 (43.9%)	69 (39.0%)	82 (42.3%)	64 (53.8%)	0.036*
Diabetes mellitus no. (%)	56 (11.4%)	18 (10.2%)	19 (9.8%)	19 (16.0%)	0.201
Infection no. (%)	43 (8.8%)	10 (5.6%)	17 (8.8%)	16 (13.4%)	0.131
**Therapy**					
Glucocorticoids no. (%)	370 (75.5%)	85 (48.0%)	175 (90.2%)	110 (92.4%)	**<0.001^#^**
Immunosuppressive agents no. (%)	364 (74.3%)	100 (56.5%)	162 (83.5%)	102 (85.7%)	**<0.001^#^**
ACEI/ARB no. (%)	387 (79.0%)	137 (77.4%)	153 (78.9%)	97 (81.5%)	0.695
VD supplementation no. (%)	451 (92.0%)	146 (82.5%)	186 (95.9%)	119 (100.0%)	<0.001*

SBP, systolic blood pressure; DBP, diastolic blood pressure; Anti-PLA2R Ab, serum anti-phospholipase A2 receptor antibody; TC, serum total cholesterol; TG, serum triglycerides; HDL-C, serum high-density lipoprotein cholesterol; LDL-C, serum low-density lipoprotein cholesterol; eGFR, estimated glomerular filtration rate; IgG, serum immunoglobulin G; IgA, serum immunoglobulin A; IgM, serum immunoglobulin M; C3, serum complement 3; C4, serum complement 4; 25(OH)D, 25-hydroxyvitamin D; ACEI, angiotensin-converting enzyme inhibitor; ARB, angiotensin receptor inhibitor; VD, vitamin D. Urinary protein: 3.5–8.0 g/L (≥3.5 and ≤8.0).

*There was a statistical difference between the groups.

^#^There was no statistical difference between Group 2 (urinary protein: 3.5–8.0 g/L) and Group 3 (urinary protein > 8.0 g/L).

Data were presented as the mean ± standard, the median with interquartile range or counts and percentages. Bold values denote statistical significance at the *P* < 0.05 level.

### Correlations between baseline 25 (OH)D level and clinical parameters

**FIGURE 2:**
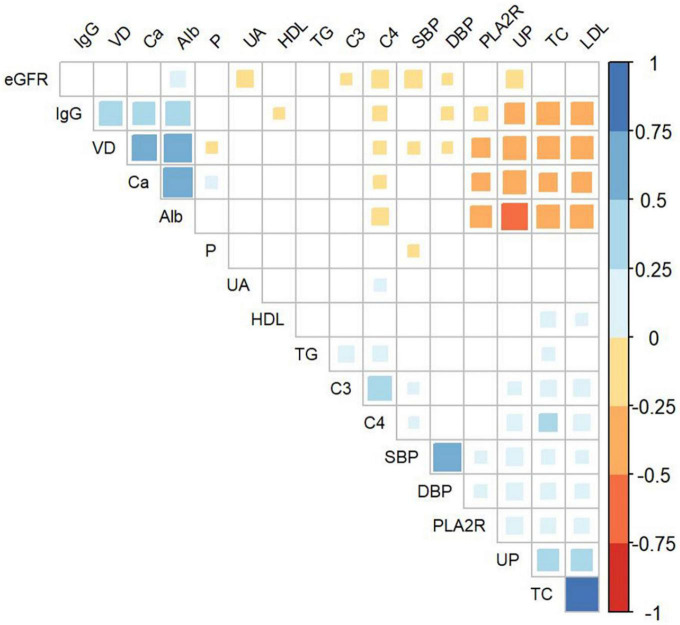
Association of baseline 25(OH)D and clinical parameters in all enrolled patients. VD, serum 25-hydroxyvitamin D; IgG, serum Immunoglobulin G; Alb, serum albumin; UA, serum uric acid; TG, serum triglyceride; C3, serum complement 3; C4, serum complement 4; SBP, systolic blood pressure; DBP, diastolic blood pressure; PLA2R, seropositivity of anti-phospholipase A2 receptor antibody; UP, urinary protein; TC, serum total cholesterol; LDL, serum low-density lipoprotein cholesterol; Ca, serum calcium; P, serum phosphate; eGFR, estimated glomerular filtration rate.

Figure 2 displays the correlations between the baseline 25(OH)D level and clinical parameters. The positive correlations between 25(OH)D level and serum albumin (*r* = 0.62, *P* < 0.001), serum calcium (*r* = 0.52, *P* < 0.001), and serum IgG (*r* = 0.44, *P* < 0.001) were observed. Meanwhile, 25(OH)D level was negatively correlated with 24 h UP (*r* = −0.43, *P* < 0.001), serum TC (*r* = −0.40, *P* < 0.001), serum LDL-C (*r* = −0.39, *P* < 0.001), serum phosphate (*r* = −0.10, *P* = 0.032), and seropositivity of anti-PLA2R Ab (*r* = −0.29, *P* < 0.001), serum C4 (*r* = −0.12, *P* = 0.008), SBP (*r* = −0.12, *P* = 0.009), and DBP (*r* = −0.09, *P* = 0.041). No significant association was observed for other clinical parameters, including TG, HDL-C, eGFR, serum C3, and uric acid.

### Associations of baseline 25(OH)D level with proteinuria and anti-PLA2R Ab level

Based on the results that the baseline 25(OH)D level was significantly discriminated between different groups of 24 h UP, further ROC curves were employed to determine the predictive value of 25(OH)D on proteinuria and the seropositivity of anti-PLA2R Ab. Baseline 25(OH)D level showed an AUC of 0.780 for predicting UP ≥ 3.5 g/day with a 72.5% sensitivity and 73.4% specificity (Supplementary Figure 1A), and an AUC of 0.758 for predicting UP > 8 g/day with a 77.3% sensitivity and 64.2% specificity (Supplementary Figure 1B). Accordingly, the optimal cutoff predictive value of baseline 25(OH)D for differentiating the diagnosis of NS was 19.55 nmol/L (AUC = 0.79), as calculated by obtaining the best Youden index (Supplementary Figure 1C). In addition, the optimal cutoff value of 25(OH)D level for predicting seropositivity of anti-PLA2R Ab was 20.05 nmol/L (AUC = 0.672) (Supplementary Figure 1D). By further dividing patients into strata based on the above cutoff values, we found that the lower level of baseline 25(OH)D was associated with an increased risk for patients with PMN presenting NS (OR 8.0, 95% CI 5.3, 12.1, *P* < 0.001). After adjusting for sex, age, and MBP, the associations between 25(OH)D level and the diagnosis of NS were still significant with an OR of 7.9 (95% CI 5.1, 12.2, model 1 in Table 2). After extensive adjustment for sex, age, MBP, anti-PLA2R Ab, and eGFR, patients with 25(OH)D with an optimal cutoff value of ≤19.55 nmol/L still had 6.8 times higher risk for the diagnosis of NS compared to those with 25(OH)D > 19.55 nmol/L (model 2, 95%CI 4.4, 10.7, *P* < 0.001) (Table 2). Moreover, compared with patients with 25(OH)D with a cutoff value >20.05 nmol/L, patients with 25(OH)D ≤ 20.05 nmol/L had 3.2 times higher likelihood of positive anti-PLA2R Ab (95%CI 2.2, 4.7, *P* < 0.001). This increased risk persisted in the lower 25(OH)D, even after adjustment for sex, age, and MBP (OR 3.1, 95%CI 2.1, 4.5, *P* < 0.001, model 1 in Table 3). When adjusting for sex, age, MBP, 24 h UP, and eGFR, patients with 25(OH)D ≤ 20.05 nmol/L still had 2.4 times higher probability for seropositivity of anti-PLA2R Ab (model 2, 95%CI 1.6, 3.7, *P* < 0.001) (Table 3).

**TABLE 2:** Predicted performance of baseline 25(OH)D for patients with PMN who had the nephrotic syndrome.

Variables	Baseline 25(OH)D (nmol/L)		*P*-value
	**≤19.55** **(*n* = 274)**	**>19.55** **(*n* = 216)**	
Patients with nephrotic syndrome, no. (%)	225 (82.1%)	79 (36.6%)	<0.001
Unadjusted ORs	8.0 (5.3,12.1)	Reference	<0.001
Adjusted ORs, adjusted for			
Model 1: age, gender and MBP	7.9 (5.1,12.2)	Reference	<0.001
Model 2: model 1 + anti-PLA2R Ab + eGFR	6.8 (4.4,10.7)	Reference	<0.001

Odds ratio (OR) and 95% confidence intervals were derived from logistic regression analyses. Model 1: unadjusted. Model 2: adjusted for age, gender, and MBP. Model 3: Model 2 plus anti-PLA2R Ab and eGFR. MBP, mean blood pressure; eGFR, estimated glomerular filtration rate; Anti-PLA2R Ab, anti-phospholipase A2 receptor antibody. A two-tailed *P* < 0.05 was considered statistically significant.

**TABLE 3:** Predicted performance of baseline 25(OH)D for seropositivity of the anti-PLA2R Ab in patients with PMN.

Variables	Baseline 25(OH)D (nmol/L)		*P*-value
	**≤20.05** **(*n* = 279)**	**>20.05** **(*n* = 211)**	
Patients with seropositivity of the anti-PLA2R Ab, no. (%)	201 (72.0%)	94 (44.5%)	<0.001
Unadjusted ORs	3.2 (2.2,4.7)	Reference	<0.001
Adjusted ORs, adjusted for			
Model 1: age, gender and MBP	3.1 (2.1,4.5)	Reference	<0.001
Model 2: model 1 + 24 h urinary protein + eGFR	2.4 (1.6,3.7)	Reference	<0.001

Odds ratio (OR) and 95% confidence intervals were derived from logistic regression analyses. Model 1: unadjusted. Model 2: adjusted for age, gender, and MBP. Model 3: Model 2 plus 24-h urinary protein and eGFR. Anti-PLA2R Ab, anti-phospholipase A2 receptor antibody; MBP, mean blood pressure; eGFR, estimated glomerular filtration rate. A two-tailed *P* < 0.05 was considered statistically significant.

### Comparisons of parameters between groups with different outcomes

The final follow-up cohort was composed of 146 patients diagnosed with PMN concurrent with NS by biopsy and with complete follow-up data. During the median 19.8 (IQR 12.5, 30.5) month follow-up, 107 patients reached remission, including 60 (56.1%) with CR and 47 (43.9%) with PR. Accordingly, patients were divided into two groups, namely, the remission group (CR + PR) (*n* = 107) and the no remission group (NR) (*n* = 39). As shown in Table 4, the titer of serum anti-PLA2R Ab, the proportion of male patients, and the incidence of infection was significantly increased, while levels of serum albumin, baseline 25(OH)D, follow-up 25(OH)D, △25(OH)D levels, and serum calcium were significantly decreased in NR compared to the remission group (*P* < 0.05).

**TABLE 4:** Characteristics of patients with nephrotic syndrome according to different outcomes.

	Total	CR + PR	NR	*P*-Value
	***n* = 146**	***n* = 107**	***n* = 39**	
**Demographic characteristics**				
Male sex, no. (%)	99 (67.8%)	66 (61.7%)	33 (84.6%)	**0.009**
Age, y	54.0 (42.8,63.0)	54.0 (39.0,63.0)	56.0 (48.0,65.0)	0.127
**Clinical characteristics**				
SBP, mmHg	134.0 (124.0,145.0)	134.0 (124.0,145.0)	131.0 (123.0,141.0)	0.402
DBP, mmHg	84.5 (77.0,91.3)	85.0 (78.0,93.0)	80.0 (74.0,90.0)	0.269
MBP, mmHg	100.3 (91.9,109.1)	101.0 (93.3,109.3)	98.3 (90.7,107.0)	0.271
**Laboratory characteristics**				
Anti-PLA2R Ab, RU/mL	79.7 (21.1,218.2)	56.9 (15.4,155.0)	160.7 (71.1,494.2)	**<0.001**
Albumin, g/L	20.5 ± 4.2	21.0 ± 4.2	19.1 ± 4.1	**0.012**
Urinary protein, g/d	7.71 (5.29,10.58)	7.69 (5.52,10.52)	7.77 (5.19,11.15)	0.810
Baseline 25(OH)D, nmol/L	12.95 (3.75,19.43)	14.50 (7.60,20.10)	10.30 (3.75,16.60)	**0.020**
Follow-up 25(OH)D, nmol/L	51.00 (34.35,70.65)	57.90 (41.50,75.30)	23.30 (12.60,42.8)	**<0.001**
Δ25(OH)D, nmol/L	35.28 (16.78,58.18)	44.40 (28.40,62.90)	10.80 (1.00,33.60)	**<0.001**
TC, mmol/L	7.74 (6.30,9.13)	7.94 (6.36,9.28)	7.21 (6.21,8.48)	0.115
TG, mmol/L	2.13 (1.46,2.84)	2.12 (1.50,2.96)	2.20 (1.39,2.71)	0.808
HDL-C, mmol/L	1.40 (1.16,1.64)	1.42 (1.17,1.66)	1.31 (1.02,1.53)	0.193
LDL-C, mmol/L	4.87 (3.87,5.72)	5.01 (3.94,6.06)	4.46 (3.67,5.52)	0.095
Serum calcium, mmol/L	1.91 ± 0.01	1.93 ± 0.13	1.86 ± 0.11	**0.002**
Serum phosphorus, mmol/L	1.20 ± 0.02	1.20 ± 0.20	1.18 ± 0.23	0.639
Serum creatinine, μmol/L	74.00 (62.23,88.68)	72.40 (60.20,86.70)	77.60 (63.40,95.80)	0.119
eGFR, ml/min/1.73 m^2^	95.14 (79.58,108.90)	96.44 (81.18,109.67)	91.51 (74.31,105.94)	0.240
Uric acid, μmol/L	374.4 ± 104.1	373.5 ± 101.9	376.7 ± 111.2	0.872
Serum IgG, g/L	4.90 (3.74,6.51)	5.06 (3.80,6.54)	4.59 (3.53,6.25)	0.293
Serum IgA, g/L	1.96 (1.42,2.55)	1.96 (1.49,2.62)	1.90 (1.29,2.54)	0.647
Serum IgM, g/L	0.98 (0.65,1.36)	0.96 (0.66,1.36)	1.08 (0.64,1.41)	0.744
Serum C3, g/L	1.0 ± 0.2	1.0 ± 0.2	0.9 ± 0.2	0.365
Serum C4, g/L	0.26 (0.22,0.31)	0.27 (0.23,0.31)	0.26 (0.21,0.31)	0.748
**Comorbid disease**				
Hypertension no. (%)	75 (51.4%)	53 (49.5%)	22 (56.4%)	0.462
Diabetes mellitus no. (%)	20 (13.7%)	14 (13.1%)	6 (15.4%)	0.721
Infection no. (%)	13 (8.9%)	6 (5.6%)	7 (17.9%)	**0.021**
**Therapy**				
Glucocorticoids no. (%)	141 (96.6%)	103 (96.3%)	38 (97.4%)	0.730
Immunosuppressive agents, no. (%)	140 (95.9%)	102 (95.3%)	38 (97.4%)	0.570
ACEI/ARB no. (%)	124 (84.9%)	91 (85.0%)	33 (84.6%)	0.949
**Follow up**				
Follow-up time, m	19.8 (12.5,30.5)	20.4 (12.6,31.0)	18.2 (10.6,26.4)	0.230

CR, complete remission; PR, partial remission; NR, non-remission/response; SBP, systolic blood pressure; DBP, diastolic blood pressure; MBP, mean blood pressure; Anti-PLA2R Ab, serum anti-phospholipase A2 receptor antibody; TC, serum total cholesterol; TG, serum triglycerides; HDL-C, serum high-density lipoprotein cholesterol; LDL-C, serum low-density lipoprotein cholesterol; eGFR, estimated glomerular filtration rate; IgG, serum immunoglobulin G; IgA, serum immunoglobulin A; IgM, serum immunoglobulin M; C3, serum complement 3; C4, serum complement 4; ACEI, angiotensin-converting enzyme inhibitor; ARB, angiotensin receptor inhibitor. Δ25 (OH)D: the difference between follow-up 25(OH)D and baseline 25(OH)D. Data were presented as the mean ± standard, the median with interquartile range or counts and percentages. Bold values denote statistical significance at the *P* < 0.05 level.

### Relationships between 25(OH)D level and remission rate in patients with PMN who had nephrotic syndrome

To substantiate our findings, we employed ROC analysis to evaluate the predictive value of baseline and follow-up 25(OH)D levels on remission rate (CR or PR). The follow-up 25(OH)D level showed a favorable predictive ability for remission (CR or PR) with an AUC of 0.844 (sensitivity = 94.4%, specificity = 59.0%), which was followed by △25(OH)D (AUC = 0.817, sensitivity = 84.1%, specificity = 69.2%) (Supplementary Figures 2A, B). The optimal cutoff values for 25(OH)D levels during follow-up and △25(OH)D for predicting remission were 26.35 and 22.00 nmol/L, respectively. Meanwhile, the AUC of baseline 25(OH)D for predicting remission was 0.625 with a sensitivity of 54.2% and a specificity of 71.8% (Supplementary Figure 2C).

**FIGURE 3:**
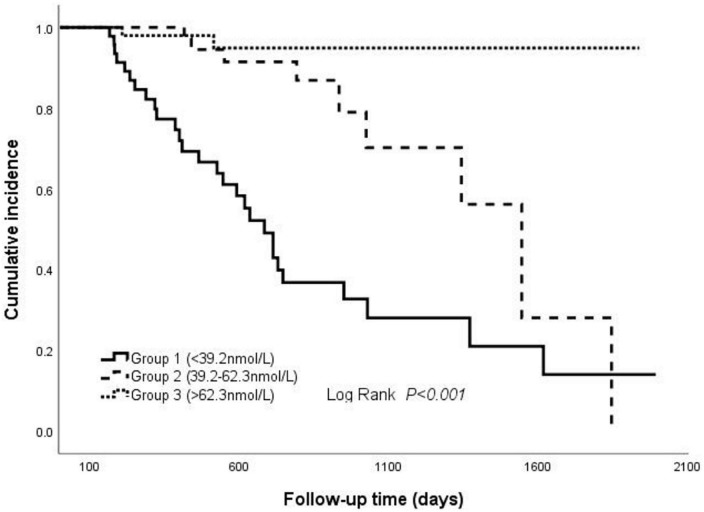
Cumulative incidence of CR and PR between different groups shown by the Kaplan–Meier survival curve. The groups were stratified according to the tri-sectional quantiles of follow-up 25(OH)D levels. The difference among the three groups was tested by a log-rank test (*P* < 0.001).

We further divided the cohort into three groups according to the tertiles of follow-up 25(OH)D levels. The Kaplan–Meier survival analysis demonstrated that the higher level of 25(OH)D during follow-up had a higher possibility of remission than lower levels (log-rank test, *P* < 0.001) (Figure 3). Moreover, the association between the follow-up 25(OH)D and risks for NR was determined by the Cox proportional hazard model (Table 5). In unadjusted models, when compared with the third tertile (≥62.3 nmol/L), the second tertile (Group 2: 39.2–62.3 nmol/L) and the first tertile (Group 1: <39.2 nmol/L) were associated with 4.97 times and 19.16 times higher likelihoods for NR, respectively. The associations remained statistically significant after adjusting for age, gender, MBP, 24 h UP, anti-PLA2R Ab, serum albumin, and serum C3 in multivariate analysis (Group 2: HR 4.90, 95% CI 1.02, 23.53 *P* = 0.047, Group 1: HR 17.52, 95% CI 4.04, 76.03 *P* < 0.001; vs. Group 3).

**TABLE 5:** Risk factors of non-remission in PMN determined by univariate and multivariate Cox regression.

	Univariate analysis		Multivariate analysis	
	**HR (95% CI)**	***P*-value**	**HR (95% CI)**	***P*-value**
**Gender**				
Female				
Male	2.76 (1.15,6.63)	**0.023**	3.25 (1.11,9.51)	**0.031**
**Age**				
<55 years				
≥55 years	1.03 (0.54,1.95)	0.936	1.21 (0.58,2.51)	0.618
MBP	0.99 (0.96,1.02)	0.395	0.99 (0.96,1.02)	0.435
**Serum anti-PLA2R Ab**				
<20 RU/ml				
≥20 RU/ml	1.33 (0.58,3.03)	0.500	1.41 (0.67,3.01)	0.51
UP				
≤8 g/day				
>8 g/day	0.97 (0.51,1.85)	0.934	1.42 (0.67,3.01)	0.359
**Follow-up 25(OH)D**				
Group 1 (<39.2)	19.16 (4.56,80.54)	<0.001	17.52 (4.04,76.03)	**<0.001**
Group 2 (39.2–62.3)	4.97 (1.07,23.08)	0.041	4.90 (1.02,23.53)	**0.047**
Group 3 (≥62.3)	Reference	1	Reference	1
Serum albumin	0.91 (0.84,0.98)	**0.023**	0.98 (0.89,1.08)	0.692
TC	0.93 (0.80,1.07)	0.307		
HDL	0.75 (0.33,1.72)	0.494		
LDL	0.84 (0.68,1.05)	0.126		
eGFR	0.99 (0.98,1.00)	0.108		
Serum IgG	0.89 (0.75,1.06)	0.182		
Serum C3	0.12 (0.03,0.55)	**0.006**	0.20 (0.04,1.15)	0.072
Serum C4	0.53 (0.01,40.17)	0.773		

Multivariate analysis was adjusted for age, gender, MBP, 24-h urinary protein, serum anti-PLA2R Ab, serum albumin, and serum C3. MBP, mean blood pressure; Anti-PLA2R Ab, anti-phospholipase A2 receptor antibody; UP, urinary protein; TC, serum total cholesterol; HDL-C, serum high-density lipoprotein cholesterol; LDL-C, serum low-density lipoprotein cholesterol; eGFR, estimated glomerular filtration rate; IgG, serum immunoglobulin G; C3, serum complement 3; C4, serum complement 4. Bold values denote statistical significance at the *P* < 0.05 level.

## Discussion

Our study confirmed the association between 25(OH)D and clinical parameters of disease activity and treatment response in PMN. First, we found a significant difference in baseline 25(OH)D among patients with low levels of proteinuria (<3.5 g/day), nephrotic proteinuria (3.5–8 g/day), and massive proteinuria (>8 g/day). Second, the lower level of baseline 25(OH)D was associated with an increased risk for the occurrence of NS and a higher likelihood of a positive anti-PLA2R Ab in patients with PMN. Then, we focused on the correlation between follow-up 25(OH)D and clinical outcomes and found that 25(OH)D significantly differentiated between the remission group (CR or PR) and the NR group in the follow-up cohort. Furthermore, lower levels of 25(OH)D during follow-up were demonstrated as an independent risk factor for NR even after adjusting age, gender, MBP, 24 h UP, anti-PLA2R Ab, serum albumin, and serum C3.

Vitamin D, an essential substance for the development of the body, is known for playing a key role in the musculoskeletal system and the metabolism of calcium and phosphorus (20, 21). 25(OH)D, acting as the metabolic product of VD from the liver, was the dominant form of existence in the circulatory system and was used for assessing VD state in clinical practice (7). Epidemiological studies have documented a high prevalence of VD deficiency as a global problem (19, 22). VD was synthesized mainly in the skin after being exposed to ultraviolet light, and a small amount was obtained through diet, so outdoor exercise, sun exposure, nutritional status, seasonal alternation, dietary habits, and other factors could affect serum concentrations of 25(OH)D independently of the renal alterations (23). Besides, VD deficiency had a high prevalence in patients with kidney disease, the reasons for which included impaired VD function due to the action of 1α-hydroxylase, which declined at the same pace as reduced nephron mass, and VD-binding protein (DBP), which was less represented in the renal proximal tubule (13). In addition, a growing body of evidence suggested that serum 25(OH)D levels were related to the activity and severity of diseases (24, 25). One major recent study found that VD deficiency increased all-cause mortality and that a concentration of 25(OH)D > 50 nmol/L could decrease the risk of death (26). Lin TC et al. found that serum 25(OH)D levels were lower in patients with LN than in those without renal lesions. Despite the absence of nephrotic lesions and urinary protein loss, lupus patients with disease activity had lower levels of 25(OH)D, and serum 25(OH)D levels were inversely correlated with disease activity (24). Thus, the potential effects of VD deficiency on kidney diseases need more in-depth studies.

Our results suggested that VD deficiency may be one of the critical mechanisms in the pathological process of PMN. Previous studies have demonstrated that 25(OH)D deficiency is related to podocyte injury, and VD supplement treatment can relieve proteinuria by restoring the nephrin signaling pathway in podocytes in diabetic nephropathy mouse models (14). Moreover, VD could inhibit RAAS and relieve interstitial fibrosis (14). In LN, 25(OH)D reduced aberrant autophagy to protect podocytes from injury caused by autoantibodies (27). More recently, Ristov MC et al. identified the VD signaling pathway as podocyte protective and as counteracting their dedifferentiation (28). Agrawal S et al. summarized that VD activated the VD receptor (VDR) and ameliorated podocyte injury in experimental models and cultured human podocytes. One potential mechanism was that VDR played significant roles in podocyte signaling and the maintenance of podocyte health (29). Altogether, low 25(OH)D levels may be involved in the podocyte injury and then the kidney damage in MN, considering MN as one of the major and typical podocytopathies. Besides, additional studies have suggested that VD could regulate immunity by targeting immune-related cells, such as T regulatory (Treg) cells, helper T cells (Th)17 and Th9 cells, B cells, and antigen-presenting cells, including dendritic cells and monocytes (12). The VDR was widely identified in activated T and B cells and monocytes (30, 31). Tsoukas CD et al. suggested that 1,25(OH)2D3 inhibited activated T-cell proliferation *via* suppressing interleukin-2 (IL-2), a lymphokine-promoting T-cell growth (30). Moreover, 1,25(OH)2D3 had inhibitory effects on effector functions of T and B cells, switched memory B cells, and plasma cells (31, 32). However, 1,25(OH)2D3 could promote the function of monocytes in antigen presentation, which presented the opposite effects on immunosuppression. Thus, the balance between positive and negative immunoregulation of 1,25(OH)2D3 played an important role in immunity (31). Furthermore, it was reported that VD regulated the inducement of regulatory T (Treg) cells, and VD deficiency was found to influence the function of Treg cells among pregnant women (33, 34). In addition, PMN was known as imbalanced immune regulation, including changes in helper T (Th) cell subsets, decreased Treg cells, and increased Th17-Treg ratio (35–37). The changes in immunoglobulin heavy chain (IGH) repertoire in B cell receptors (BCR) were observed in patients with PMN (38). Rituximab (RTX), an agent targeting CD20 + B cells, was recommended as the first-line treatment option for PMN, which further demonstrated the key role of B cells in the pathogenesis of PMN (39, 40). Treg cells were also a useful predictor for RTX treatment (36). Hence, VD might contribute to regulating immunological reactions in the pathogenesis of PMN. Further studies would be required for the mechanism of VD deficiency needed to be better understood in the context of podocyte biology and pathological process of MN.

In our study, baseline 25(OH)D level was negatively associated with 24 h UP and showed predictive ability for UP ≥ 3.5 g/day and UP > 8 g/day. The findings were further supported by the multivariate logistic regression analysis, which showed that patients with 25(OH)D ≤ 19.55 nmol/L had 6.8 times higher risk for the incidence of NS compared to those with 25(OH)D > 19.55 nmol/L in a fully adjusted model. According to the KDIGO 2021 glomerular diseases guidelines, 24 h UP was not only a solid predictor of the clinical outcome but also a favorable indicator of risk stratification and risk-based treatments in PMN (16). Previous studies have demonstrated 25(OH)D levels were independently associated with albuminuria in patients with CKD (41–44). A series of studies explained that VD suppressed renin transcription, inhibiting the RAAS, and ultimately leading to a reduction in proteinuria through hemodynamic and non-hemodynamic pathways (45, 46). However, in the circulation, most of 25(OH)D was bound with DBP, some with serum albumin, and only less than 1% existed in a dissociative form (7). Increasing levels of proteinuria may perpetuate VD deficiency due to direct loss of DBP-bound 25(OH)D in the urine (47). Altogether, there may be a synergistic interplay between low 25(OH)D level and proteinuria, leading to a vicious cycle for progressive deterioration of renal function (13). Moreover, in several randomized controlled trials, active VD supplementation has been suggested to reduce the degree of proteinuria in patients with proteinuric kidney disease, and hence recent guidelines support 25(OH)D supplementation in CKD due to the anti-proteinuric effect (46, 48). Coupled with our observation that the lower follow-up 25(OH)D level was an independent risk factor for NR, patients with PMN who had low 25(OH)D levels should receive more attention in intensive VD supplementation. Additionally, our data suggested that patients with PMN may benefit from the 25(OH)D levels that increased by 22.00 nmol/L and reached at least 62.3 nmol/L after VD treatment to achieve a high probability of remission outcome.

Primary membranous nephropathy was a typical organ-specific autoimmune disease, arising from a loss of normal immune tolerance to podocyte antigens with the formation of disease-causing antibodies that resulted in a pathognomonic pattern of injury in glomeruli (40, 49). PLA2R, a landmark podocyte antigen, was associated with 70–80% PMN (2). Serum anti-PLA2R Ab, as the product of the immune reaction, aided in disease diagnosis, monitoring treatment response, and evaluating the prognosis of PMN (3, 4, 50). The decline of anti-PLA2R Ab levels has been proven to predict immunological remission (51). According to the 2021 KDIGO guidelines, serum anti-PLA2R Ab has been well-applied as a valuable biomarker for evaluating the risk of progressive loss of kidney function and initiating adapted treatment for each patient in clinical practice (52). In the study, we found that baseline 25(OH)D level was negatively correlated with seropositivity of anti-PLA2R Ab. ROC curve analysis demonstrated the ability of baseline 25(OH)D to discriminate patients with seropositivity of anti-PLA2R Ab (sensitivity 68.1%, specificity 60.0%), and the cutoff value was 20.05 nmol/L (AUC = 0.672). Logistic regression analysis further verified that a lower level of 25 (OH)D was related to the higher likelihood of seropositivity of anti-PLA2R Ab (OR 2.4 95%CI 1.6, 3.7, *P* < 0.001) in the fully adjusted model. Our findings supplied evidence for the association of VD and B cell homeostasis since autoantibodies generated by autoreactive B cells were a key driver in the immunopathogenesis of MN (40). Numerous studies have reported that active VD suppresses the proliferation of activated B cells, generation of switched memory B cells, differentiation of antigen-secreting plasma cells, and Ig secretion (32, 53). VD also induced apoptosis of activated B cells (32, 53). Moreover, declined serum 25(OH)D was associated with many autoimmune diseases, such as systemic lupus erythematosus (SLE). VD plays an important role in maintaining B cell homeostasis, and the deficiency might contribute to B cell hyperactivity in SLE (32). Hence, we hypothesized that 25(OH)D might participate in the production of anti-PLA2R Ab *via* exerting effects on B cells, which needed further well-designed studies to explore. In addition, when related to the clinical outcome, lower baseline and follow-up 25(OH)D levels were associated with a significantly lower probability to achieve remission during follow-up. Combined with our results, VD supplementation may be important in the treatment of B cell-mediated autoimmune disorders. In addition, supplementation of VD to at least a level of 20.05 nmol/l might have a greater likelihood of getting a negative serum anti-PLA2R Ab and therefore achieving immunological remission.

There were several limitations to our study. First, many immune-related indexes, including the count and proportion of T and B cell subtypes, were not routine tests in clinical practice and were lacking in the analysis due to the nature of this retrospective study. Second, VD supplementation was prescribed to patients who had VD insufficiency (<30 ng/ml) and dynamically adjusted by measurement of serum 25(OH)D to ensure a sufficient VD status in accordance with the guidelines in the current study (18, 19). Further large, randomized controlled, prospective studies that take the accurate dose and duration of VD supplementation into consideration when relating to remission outcomes are necessary to better elucidate the efficacy of VD supplementation on PMN. Moreover, although our study identified the association between VD and clinical parameters and treatment responses, whether VD was involved in the pathogenesis of PMN *via* immunoregulation and the potential mechanism of VD deficiency on autoantibody production and heavy proteinuria would be worth exploring in future studies.

In conclusion, we found the value of baseline 25(OH)D in identifying patients with PMN who had NS or seropositivity of anti-PLA2R Ab, and the predictive ability of follow-up 25(OH)D on remission outcomes. The lower level of 25(OH)D during follow-up was demonstrated as an independent risk factor for NR after adjusting for important clinical confounders. Our data shed light on the indispensable role of VD detection and timely supplementation in MN.

## Data availability statement

The raw data supporting the conclusions of this article will be made available by the authors, without undue reservation.

## Ethics statement

The studies involving human participants were reviewed and approved by the Ethics Committee of the First Affiliated Hospital of Nanjing Medical University. The patients/participants provided their written informed consent to participate in this study.

## Author contributions

SD and YY designed the research. SC contributed to the writing. SC, MZ, LJ, and CC conducted the research. SC and FL analyzed the data and performed the statistical analysis. LG, RS, YX, ZH, CZ, BZ, and HM reviewed the manuscript. CX conceived, coordinated the study, and had responsibility for its final content. YY was the guarantor of this study, who had complete access to all the data in the study and took ultimate responsibility for the study design and integrity of data analysis. All authors read the final manuscript and approved the submission.
