# Supplementary material for: Serum 25-hydroxyvitamin D as a predictive biomarker of clinical outcomes in patients with primary membranous nephropathy
Source: Front Nutr. 2023 Apr 25;10:1171216. doi: 10.3389/fnut.2023.1171216 (PMC10167293; doi:10.3389/fnut.2023.1171216)
Supplement: Supplementary file 1 [file Data_Sheet_1.pdf]

Supplementary FIGURE 1

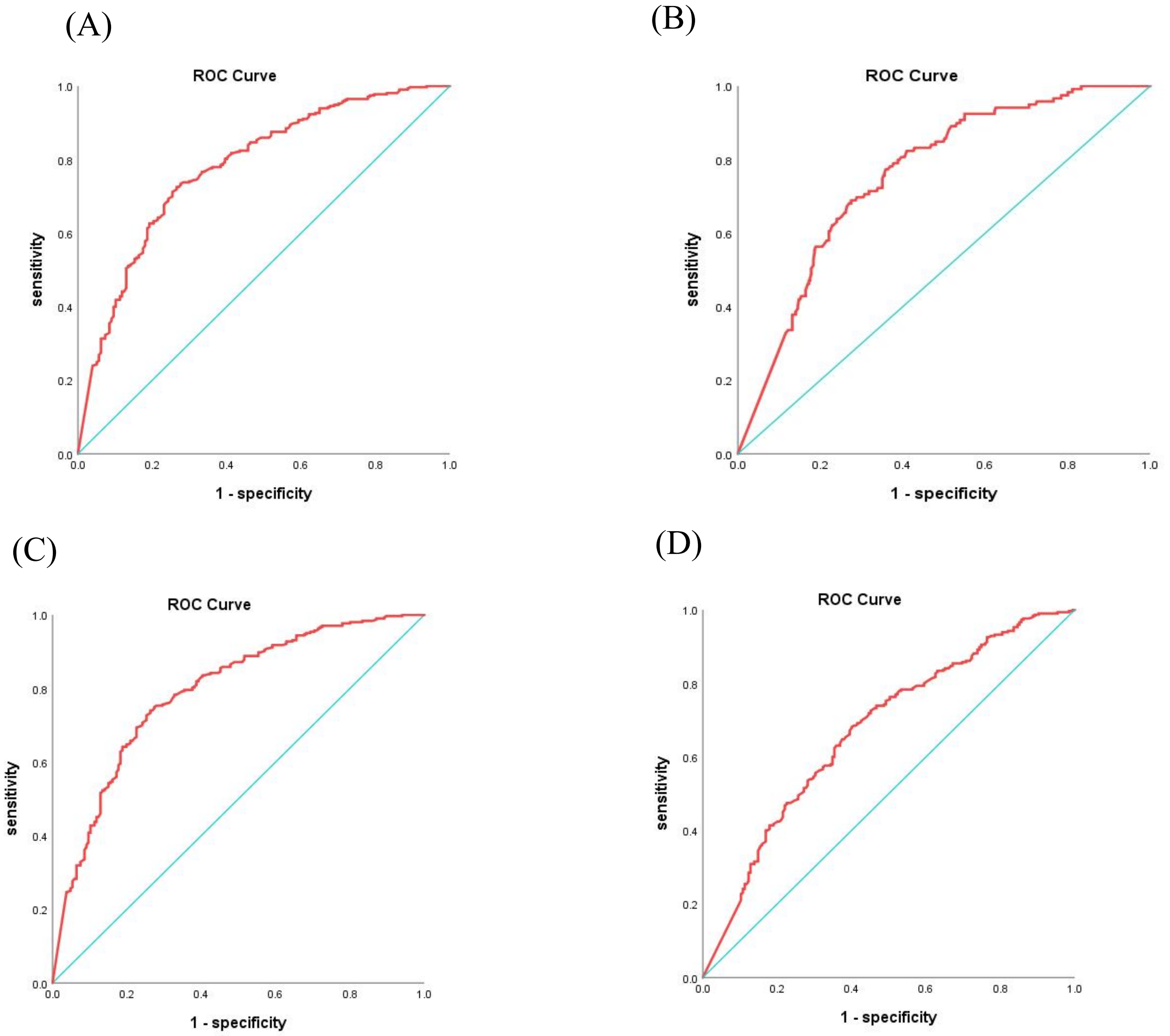

Supplementary FIGURE 2

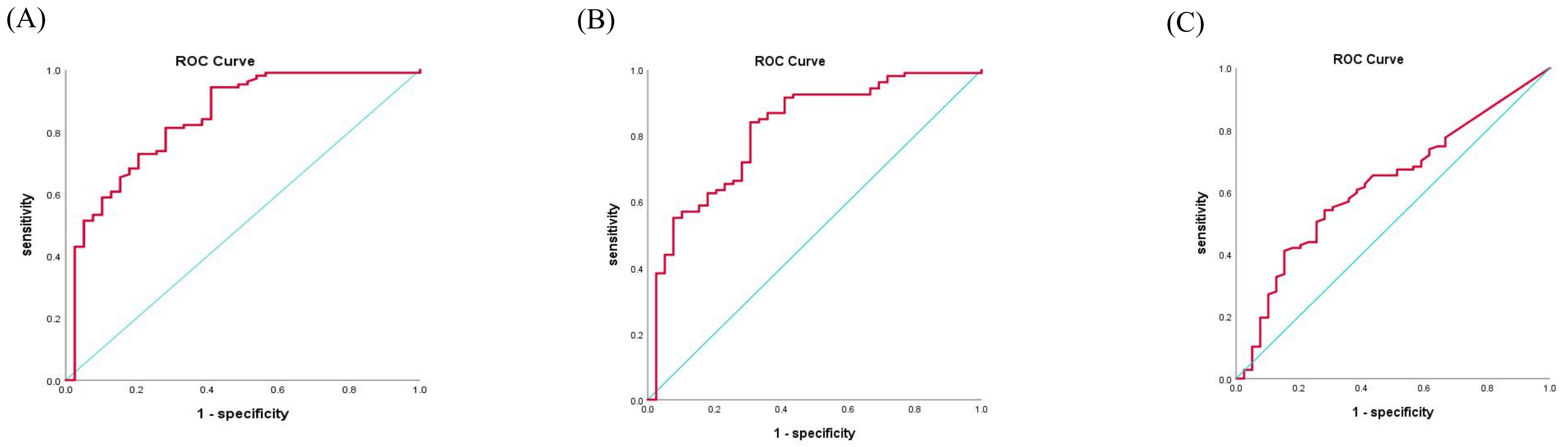

Supplementary Figure legends

Supplementary Figure 1. Association of baseline 25(OH)D and the levels of proteinuria in all enrolled patients. A. Predictive performance of baseline 25(OH)D for urinary protein ⩾ 3.5g/d evaluated by ROC curves (AUC=0.780). B. Predictive performance of baseline 25(OH)D for urinary protein>8g/d evaluated by ROC curves (AUC=0.758). C. Predictive performances of baseline 25(OH)D for patients presenting with nephrotic syndrome evaluated by ROC curves (AUC=0.790). D. Predictive performances of baseline 25(OH)D for the seropositivity of anti-PLA2R Ab evaluated by ROC curves (AUC=0.672). 25(OH)D, 25-hydroxyvitamin D. ROC, receiver operating characteristic. AUC, the area under the ROC curve.

Supplementary Figure 2. Prediction of 25(OH)D for treatment responses in follow-up cohort. A. Predictive performance of follow-up 25(OH)D for remission (CR or PR) evaluated by ROC curves (AUC=0.844). B. Predictive performance of △25(OH)D for remission (CR or PR) evaluated by ROC curves (AUC=0.817). C. Predictive performance of baseline 25(OH)D for remission (CR or PR) evaluated by ROC curves (AUC=0.625). 25(OH)D, 25-hydroxyvitamin D. ROC, receiver operating characteristic. AUC, the area under the ROC curve. △25(OH)D was the difference of 25(OH)D between baseline and follow-up.

Supplementary TABLE 1

Baseline serum PTH levels of patients with different levels of proteinuria.

Parameter	Total<br>n=339	Urinary protein (g/d)<br><3.5 (n=121)	 3.5-8.0 (n=131)	 >8.0 (n=87)	P Value
Serum PTH, pg/mL	30.70(22.90,44.60)	31.30(23.40,42.05)	29.40(22.80,43.20)	31.90(18.30,48.80)	0.690

PTH, parathyroid hormone.

Urinary protein:3.5-8.0g/L (≥3.5 and ≤8.0).

Data were presented as the median with interquartile range. A two-tailed P<0.05 was considered statistically significant.

Supplementary TABLE 2

Baseline serum PTH levels of NS patients with different outcomes

	Total<br>n=102	CR+PR<br>n=70	NR<br>n=32	P Value
Serum PTH, pg/mL	31.2(21.90,45.98)	30.20(17.88,40.35)	38.65(27.05,51.65)	0.017

NS, nephrotic syndrome; CR, complete remission; PR, partial remission; NR, non-remission/response, PTH, parathyroid hormone.

Data were presented as the median with interquartile range. A two-tailed P<0.05 was considered statistically significant.
